# Toward the validation of crowdsourced experiments for lightness perception

**DOI:** 10.1371/journal.pone.0315853

**Published:** 2024-12-23

**Authors:** Emily N. Stark, Terece L. Turton, Jonah Miller, Elan Barenholtz, Sang Hong, Roxana Bujack

**Affiliations:** 1 Computer, Computational, and Statistical Sciences Division, Los Alamos National Laboratory, Los Alamos, NM, United States of America; 2 Department of Psychology, Florida Atlantic University, Boca Raton, FL, United States of America; Fundación Universitaria del Área Andina, COLOMBIA

## Abstract

Crowdsource platforms have been used to study a range of perceptual stimuli such as the graphical perception of scatterplots and various aspects of human color perception. Given the lack of control over a crowdsourced participant’s experimental setup, there are valid concerns on the use of crowdsourcing for color studies as the perception of the stimuli is highly dependent on the stimulus presentation. Here, we propose that the error due to a crowdsourced experimental design can be effectively averaged out because the crowdsourced experiment can be accommodated by the Thurstonian model as the convolution of two normal distributions, one that is perceptual in nature and one that captures the error due to variability in stimulus presentation. Based on this, we provide a mathematical estimate for the sample size needed to produce a crowdsourced experiment with the same power as the corresponding in-person study. We tested this claim by replicating a large-scale, crowdsourced study of human lightness perception with a diverse sample with a highly controlled, in-person study with a sample taken from psychology undergraduates. Our claim was supported by the replication of the results from the latter. These findings suggest that, with sufficient sample size, color vision studies may be completed online, giving access to a larger and more representative sample. With this framework at hand, experimentalists have the validation that choosing either many online participants or few in person participants will not sacrifice the impact of their results.

## Introduction

Insights to human perception drawn from an online crowdsourced study rightfully raise skepticism. Traditionally, psychophysical studies are performed in highly controlled settings. This allows for a statistically powerful study, as confounding variables are minimized to the best of the experimenter’s ability. However, these studies are not always feasible or the best choice.

For example, this research was partly inspired by the constraints on conducting in-person studies during the COVID-19 pandemic. Crowdsourcing studies facilitate the acquisition of a more representative sample, in contrast to recruiting students from higher education institutions—a practice sometimes referred to as the "Sophomore Bias" [1–3] or Western, educated, industrialized, rich and democratic, WEIRD. Additionally, crowdsourced studies can achieve a substantially larger sample size, enhancing statistical power compared to traditional studies, and do so in a shorter period.

For these reasons, there has been increased interest in using crowdsource platforms to conduct perceptual studies (see [4] for an in-depth review) however using such an uncontrolled experimental platform for color perception is met with concern. Here, we present work that validates a recent, crowdsourced color perception study using a highly-controlled in-person experimental setup. This allows for a direct quantification of the added noise from crowdsourcing color perception experiments and a rough approximation of the magnitude of variability in color stimulus presentation.

The contributions of this paper are a discussion and mathematical description of the increased noise due to uncontrolled stimulus presentation in crowdsourced color perception including a framework for *a priori* sample size determination, the first empirical validation of a crowdsourced color perception study through repetition in a controlled environment, and derivation of an upper-bound on how much the presentation of achromatic color stimuli varies in a crowdsourced color perception study.

Amazon Mechanical Turk (MTurk) and other crowdsourced platforms have been leveraged in human behavioral research for over a decade, (e.g, [5]) and crowdsourcing has become an accepted approach to participant recruitment. Use of MTurk has extended to visual perception studies in the past [6–12]. Some studies have specifically sought to replicate past experimental results in order to validate the use of Mturk. Harrison et al. [13] reproduced the results of Rensink & Baldridge [14] regarding the perceived correlation in scatter plots. The crowdsourced study included 30 participants per experimental condition compared to the original, in-person study which had only 20 in total. Heer & Bostock [15] replicated the seminal investigation of graphical summaries of data originally completed by Cleveland & McGill [16]. Sample sizes for these studies are approximately equal, with 50 completing the crowdsourced experiment and 55 completing the in-person study.

Even studies relying on color presentation have been replicated using MTurk. In particular, Turton et al. [10] qualitatively replicated a previous study by [17] where participants had to select a color most similar to a specific place in a visualization using a color map. While the original study only included 12 participants, the crowdsourced replication included hundreds of participants that fell into one of three groups: women only, participants that were identified as color vision deficient (either by self-disclosure or failing a color vision task), or the typical MTurk users. By including these three groups, Turton et al. was able to identify that participants with a color vision deficiency were worse at the task [10]. This highlights the importance of testing for and excluding these participants in a crowdsourced study; however, these results suggest that with adequate precaution, MTurk can be used for color perception studies in visualization sciences.

There have been other studies that measure quantitative metrics relating to color perception using crowdsourcing, however they are not directly quantifying color perception. Witzel [18] expanded on earlier work by Hansen et al. [19] investigating the effect of memory on color appearance. The original work concluded that color perception is a top-down process when the stimuli presented are natural images (fruit) [19]. This work was done in-person with a sample size of 14. Witzel conducted a similar study using a crowdsourcing platform and a sample size of 354 for one task and 200 for a second [16]. Witzel reported a significant, but small, effect of familiarity with the stimulus (e.g. a banana is associated with yellow) and claimed the limited effect size was due to the lack of display calibration. Ultimately, this line of work suggests that when using natural images, color perception can be augmented by top-down processes.

Paramei et al. also utilized a crowdsource platform to conduct a cross-cultural color naming study in which they compared the categorization of color space between Russian speakers and established English basic color terms [20]. This study did include a calibration step in which participants were asked to adjust their RGB settings to provide more consistent stimuli across the 713 subjects. This study did find agreement with in-person studies suggesting that any minor variance in color calibration was not significant for a color classification task. While this provides validation for crowdsourced studies using non-natural stimuli (color patches) its applicability to measuring color perception is limited as the task is classification based rather than quantitative.

Woods et al compiled a thorough review of crowdsourced perception studies in a tutorial to address the challenges and work-arounds with these types of studies [4]. The majority of the studies reviewed focused on questionnaire-based research, reaction-time studies, and temporal/spatial stimuli. It was found that, with the exception of studies requiring stimuli to be presented for a short period of time, crowdsourcing is a viable solution for perception studies *in general*. However, color stimuli were specifically called out for being “one of the greatest challenges” for online studies. There are several potential workarounds suggested from others in the field. For example, one suggestion is asking participants to calibrate their screens, however this would increase the complexity of the study and might not be feasible for a study that is already complex and/or longer. Another idea was taking pictures of a common household object for post-hoc color correction, however this was provided in a personal communication and also presents additional assumptions when processing data. These work-arounds are presented to decrease the variability in color stimulus presentation (either directly or post-hoc), however Woods et al. did note that this could be achieved by “collecting data from many more participants.” There was no further guidance on how successful this work-around would be or how many is enough [4]. This is the primary question motivating the present work: to validate the claim and understand how many more participants is enough.

Studies involving color maps for scientific visualization inherently involve a color perception task [10]. Participant responses can be modeled using Thurtsone’s theory of comparative judgment [21]. This theory states perception is stochastic. A participant would perceive the strength of a given stimulus, *S*_*i*_, to be centered around the true perceived strength, *ψ*_*i*_, with some discriminal dispersion, *σ*_*disc*_,

$$Percept(S_{i})∼N(\psi_{i},\sigma_{disc}).$$ (1)

The discriminal dispersion determines how varied participants tend to perceive a given stimulus. Generally, Thurstone’s Case V is used, which states that the discriminal dispersion is constant across a set of stimuli rather than varying for each *S*_*i*_.

Studies that are crowdsourced have an inherently lower internal validity and higher noise or error in presenting stimuli. For the case of online studies involving color and properties of color, variability can stem from monitor brightness, display resolution, ambient light, user distance from monitor, or changes in the default colorimetric profile settings.

Suppose that, in a crowdsourced study, the experimenter wants to estimate the perception of a mid-gray value, defined in CIELAB as L* = 50,a* = 0,b* = 0. In a crowdsourced study, the presentation of a stimulus intended to be mid-gray will likely be variable. The stimulus intended to be *S*_L* = 50_ may be presented such that it appears a little lighter or darker than L* = 50, depending on the participant’s monitor and lighting. Assuming this error is normally distributed, the presentation of the stimulus is normally distributed,

$${S\prime }_{L^{*}=50}∼N(50,\sigma_{err}),$$ (2)

where *S*′_L*_ = 50 is the L* value of the gray actually presented to a participant in their individual setting and *σ*_*err*_ is the error associated with using a crowdsourced experimental design. In a traditional, highly controlled experimental setup, measures can be taken to minimize any error in presenting the stimuli, *σ*_*err*_≈0. When presenting the mid-gray stimulus in a controlled environment, one can be reasonably certain that,

$$S_{L^{*}=50}=50.$$ (3)

In this controlled environment, the stimulus would be perceived as a normally distributed variable about *ψ*_L* = 50_

$$Percept(S_{L^{*}=50})∼N(\psi_{L^{*}=50},\sigma_{disc}).$$ (4)

In the less controlled, crowdsourced experiment, the perception of the stimulus would be centered around *ψ*′_L* = 50_, which is itself stochastic centered at the true perceived strength

$$Percept({S\prime }_{L^{*}=50})∼N({\psi \prime }_{L^{*}=50}∼N(\psi_{L^{*}=50},\sigma_{err}),\sigma_{disc}).$$ (5)

From Eq 5 and the Central Limit Theorem, we may conclude that, with sufficient sample size, the perception of *S*′_L* = 50_ is centered around *ψ*′_L* = 50_. Therefore, on average, the perceived strength of the stimulus in the controlled experiment is the same as in the crowdsourced study

$$Percept(S_{L^{*}=50})=Percept({S\prime }_{L^{*}=50}).$$ (6)

The only meaningful difference in this framework is the measure of spread of the perceived strengths. In fact, let *σ*_*disc*_ be the discriminal dispersion, i.e., the perceptual noise as Thurstone (1927) defines it, and *σ*_*err*_ the standard deviation that comes from using an uncontrolled environment, then the *Percept*(*S*′_L* = 50_ is normally distributed with a standard deviation of $\sigma_{cs}=\sqrt{{\sigma_{err}}^{2}+{\sigma_{disc}}^{2}}$. Thus, while there is increased inherent noise, it should be possible to mitigate this by increasing sample size. In fact, the Central Limit Theorem guarantees that ​​for random variables that are independent and have the same distribution, the distribution of the normalized sample mean approaches the standard normal distribution, regardless of whether the original variables have a normal distribution. In particular, the mean of n variables is a random variable whose mean is the mean of the individual variables and whose standard deviation satisfies if one has an estimate for *σ*_*err*_^2^ and *σ*_*disc*_^2^, and a known in-person sample size *n*_*in*_, one could directly solve for a the sample size *n*_*cs*_ needed to get as accurate a parameter prediction from setting equal to each other the means of both experiments $\frac{\sigma_{cs}}{\sqrt{n_{cs}}}=\frac{\sigma_{disc}}{\sqrt{n_{in}}}$, which results in

$$n_{cs}=\frac{n_{in}{\sigma_{cs}}^{2}}{{\sigma_{disc}}^{2}}=\frac{n_{in}({\sigma_{disc}}^{2}+{\sigma_{err}}^{2})}{{\sigma_{disc}}^{2}}.$$ (7)

For instance, if the standard deviation associated with the lack of calibration, *σ*_*err*_, is twice that of the discriminal dispersion *σ*_*disc*_ and the in-person study has *n*_*in*_ = 12 participants, a crowd-sourced study of size *n*_*cs*_ = 60 should produce comparable results. If *σ*_*err*_ is three times that of the discriminal dispersion, then *n*_*cs*_ = 120 crowdsourced participants would be required for comparable power.

Thurstonian scaling has typically been applied to two-alternative forced-choice tasks (2AFC) which rely on the concurrent perception of two or more stimuli. In a crowdsourced version of this task, it is reasonable to assume that the error in presenting the stimuli would be constant across the multiple stimuli, so the error due to crowdsourcing would only be present, at most, once per trial.

The most obvious assumption here is that the error associated with crowdsourced studies is normally distributed and can therefore be averaged out with sufficient sample size. To test this assumption, and thus demonstrate the validity of crowdsourced color perception studies, we replicated a recent study investigating the existence of diminishing returns in color perception. Bujack et al. demonstrated, using crowdsourced participants, that larger color differences are perceived as smaller than the sum of the perception of smaller differences [22]. This phenomenon, *diminishing returns*, was first hypothesized by Judd [23]; however strong evidence for or against it was not possible without more recent analysis techniques [22]. To evaluate the existence of diminishing returns, Bujack et al. used the method of triads where participants were presented with three grays in line with one another and asked to select the right or left as being more different from the middle. They tested 320 triads, of which a subset of no more than 70 were shown to each participant. Enough participants were recruited through MTurk to achieve between 200 and 350 responses per triad. We replicate that study using a highly controlled experimental setup with minimal changes.

Based on our assumption that noise associated with crowdsourcing can be averaged out, we predicted that our findings will qualitatively agree with those of Bujack et al. [22]—that is, the results would provide evidence of diminishing returns, thus supporting the use of MTurk for color perception studies. Beyond a successful replication of [22], we were more concerned with the qualitative agreement between the learned models of perceived difference. This would lend support for crowdsourced perceptual studies, as it would show that the underlying perceptual processes are still observable through the increased noise.

To demonstrate this, we analyzed all of the experimental trials that were common between the two experiments and analyzed them using an updated, flexible maximum likelihood multiple difference scaling method that is able to learn second order effects [24]. This method provides an estimated perceived strength of differences, rather than just stimulus strength. We show that the perception of differences estimated from in-person data is in agreement with the scale estimated from a crowdsourced experiment.

## Method

### Materials

Bujack et al. constructed triads of gray by manipulating the difference in L* units between the standard, presented in the middle, and either test, presented on either side [22]. The difference between the middle and darker gray was assigned the label *d*_1_, while the difference between the middle and the lighter gray was assigned *d*_2_. These two values took on values between 0 and 30 in step sizes of 2.5, however, only triads that had a difference of differences *Δd* = *d*_1_−*d*_2_ = ±2.5,±5,±10 were used. These differences were applied to different standards of L* = 30,40,50,60,70.

Our stimuli were displayed using an NEC MultiSync PA271Q-BK IPS LCD monitor. The monitor had a refresh rate of 60 Hz and a pixel resolution of 2560–1440. The RGB values for our stimuli were calculated using MATLAB, which accounted for the red, green, and blue phosphors, measured by an Ocean Optics USB4000 Spectrometer, and the relative light level of each gun at every digital value, measured by a Minolta LS-110 Luminance Meter.

The RGB transformations based on our specific experimental setup revealed that we could not accurately display L* values less than 10. To avoid gamut issues and ensure the differences displayed were accurate, we only analyzed triads centered at 50 and higher, so the darkest gray presented was L* = 20; however, we collected data from all triads used in the previous study.

### Procedure

**Fig 1:**
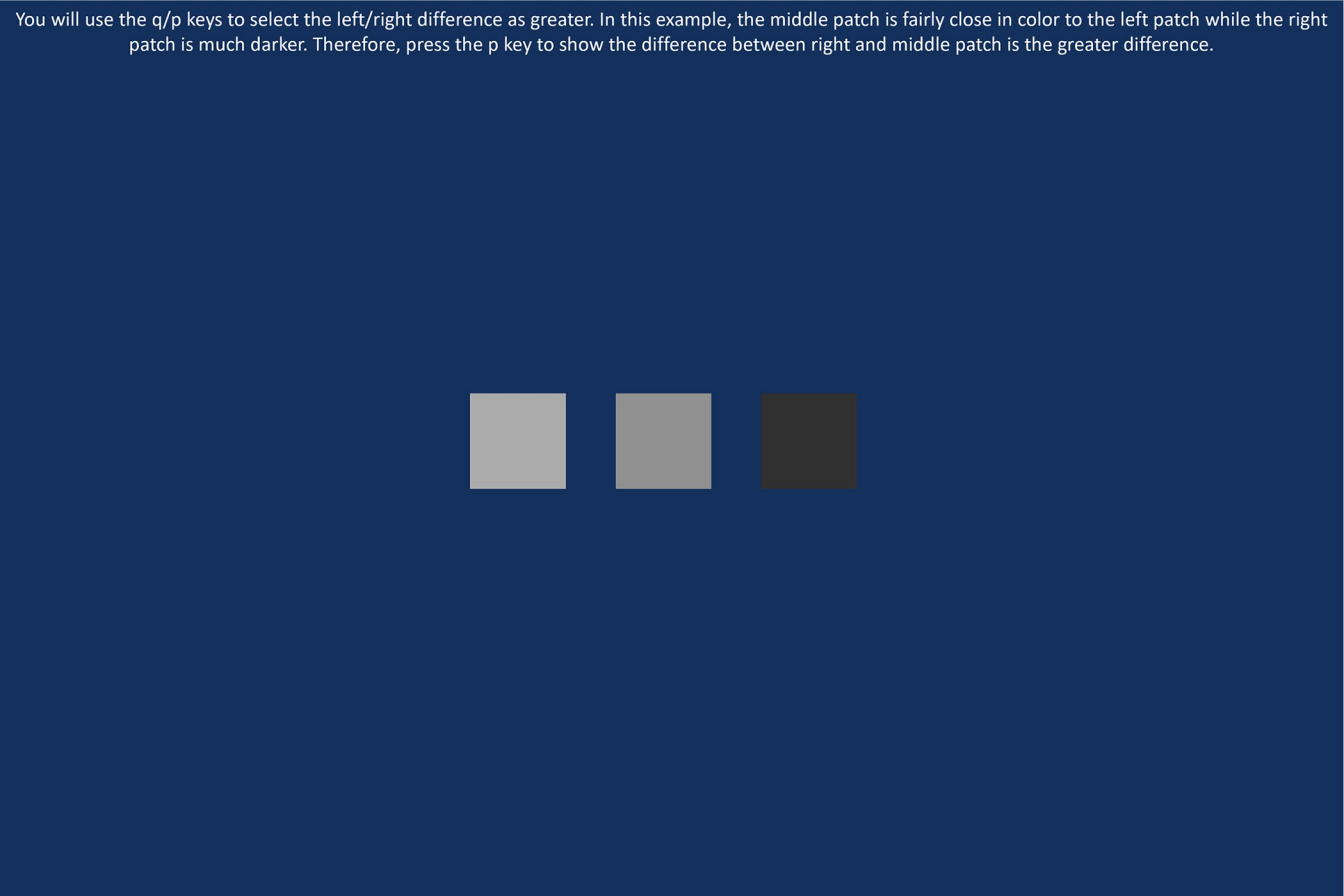
A screenshot of a training triad displayed using the Qualtrics software survey.

Participants were shown the 320 triads in 5 blocks of 64 triads each using the survey software Qualtrics. An example of a triad shown to participants can be seen in Fig 1. After each trial, they were given feedback on if they selected the correct test by seeing a gray checkmark or “x”. Participants took a two-minute rest break between each block. The order of the triads and the right and left positions of the tests were randomized. In addition to the experimental task, participants were given a test for color vision deficiency.

Participants were allowed unlimited time to respond to each triad, although most made their judgments in seconds. The shortest time taken to complete the entire study was 23.8 minutes, while the longest time was 79.1 minutes. The average time to complete the study was 34.8 minutes.

### Participants

34 participants (18 female) with ages between 18 and 32 completed the study. All participants were undergraduate or graduate students. None of the participants had known color vision deficiencies and all passed an online presentation of the Ishihara test for color blindness. Participants were treated in compliance with both the Florida Atlantic University’s Institutional Review Board (IRB) and the Los Alamos National Laboratory’s Human Subjects Research Review Board (HSRRB, IRB equivalent). Written informed consent to participate was obtained by all participants before they completed study. Written informed consent to publish results from participant data was obtained before participants completed the study. No participants withdrew consent during or after the study. One female participant was excluded from the analysis due to not understanding the task and achieving significantly below chance accuracy. The data and code for this study are publicly accessible at https://github.com/lanl/color.

## Results

### Effect of gender

A repeated measures t-test was used to check for a significant effect of gender. Across all triads, there was not a significant difference between the genders, *t(31) = -0*.*635*, *p = 0*.*530*. Data are aggregated across sex for the remainder of the analysis.

### Existence of diminishing returns

To establish the existence of diminishing returns using the in-person study, we compare the predictive power of several models of perception. First, we use maximum likelihood estimation (MLE) to learn a baseline model which scales the L* values linearly to agree with a standard deviation of 1 in the inverse normal distribution. For a detailed description of the analysis see [22] and [24]. The second model allows for a nonlinear scaling of L* to map from absolute strength to perceived strength, *ψ*, using a perceptual function, *g*(*x*). Lastly, we estimate a difference scaling function, *f*(*x*), that takes in a difference of perceived strengths and returns the perceived difference.

**Fig 2:**
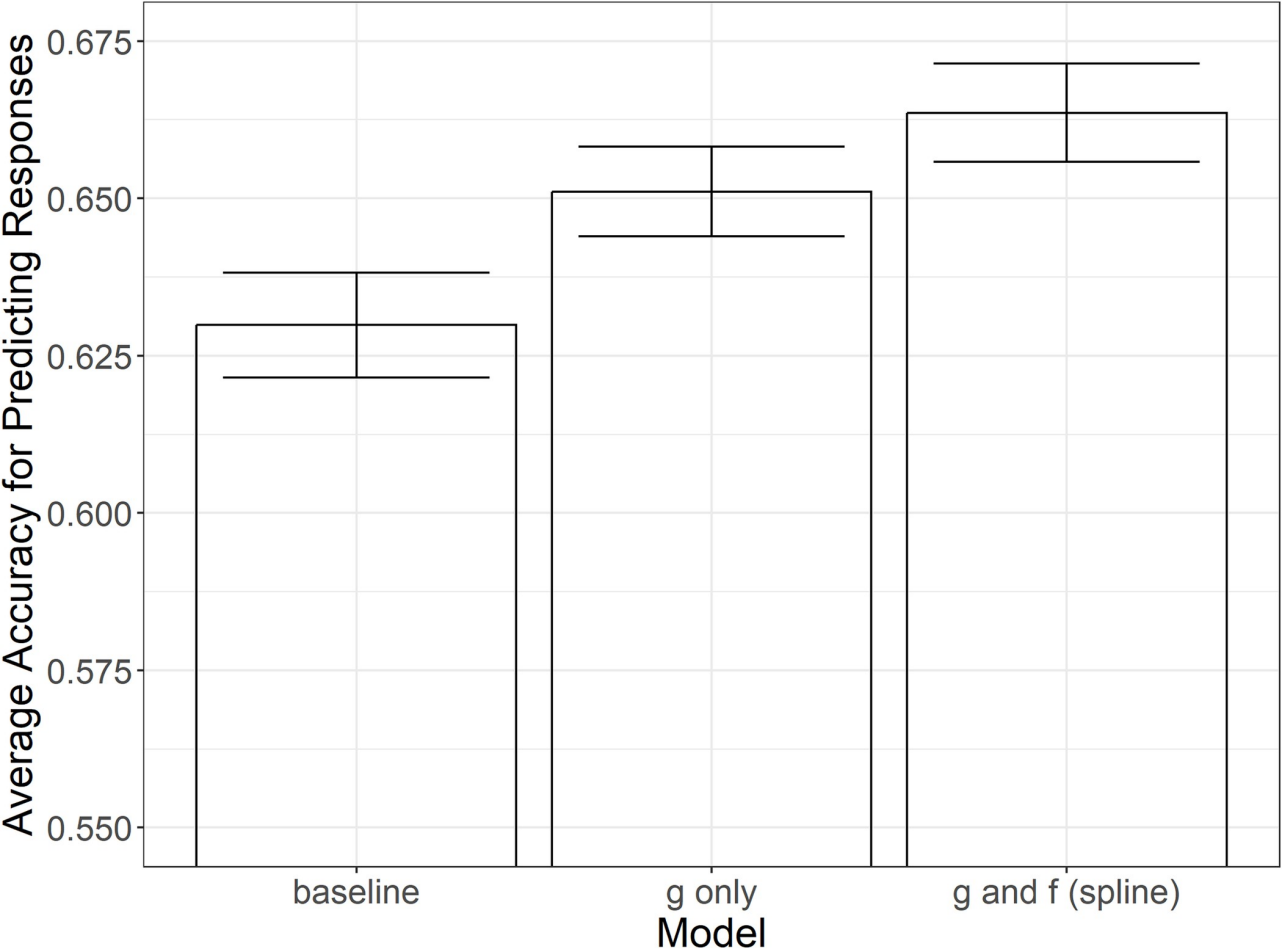
Average predictive power of the baseline condition, the model of the perceptual scale, and the model that accounts for the perceptual scale and the size of the differences.

We model the *g*(*x*) and *f*(*x*) functions using a monotonic spline function with four, nonzero control points estimated by the MLE in addition to the origin. Models were validated using leave-one-out cross-validation where each participant’s data were held out as a test set once and the MLE learned from the remaining participants. The models were evaluated based on how well they predicted the responses of the test set. The average accuracy of the models compared can be seen in Fig 2. A one-way ANOVA conducted to test for a difference in accuracy was significant, *F(2*, *294) = 18*.*74*, *p < 0*.*001*. Tukey post hoc testing found a significant difference between the accuracy of the baseline condition (*M = 0*.*630*, *SD = 0*.*042*) and the combination of the *g*(*x*) and *f*(*x*) models (*M = 0*.*664*, *SD = 0*.*040*), *p < 0*.*001*. There was a weakly significant difference between the model with only *g*(*x*) (*M = 0*.*651*, *SD = 0*.*036*) and the model with both *g*(*x*) and *f*(*x*), *p = 0*.*061*.

**Fig 3:**
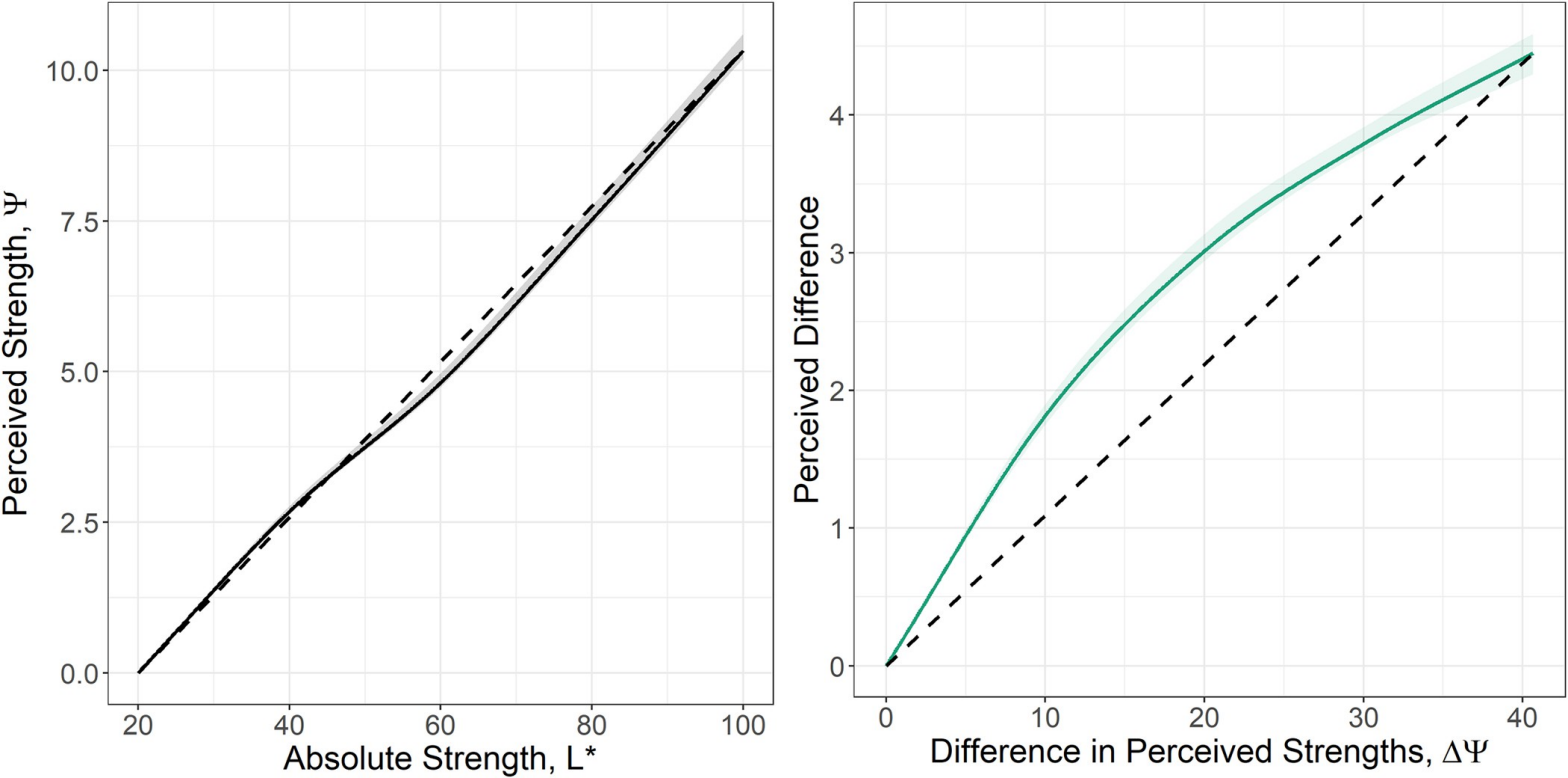
The learned models for the perceptual function (A) and the difference scaling function (B) from data collected during the in-person study.

The learned perceptual and difference scaling functions are seen in Fig 3. The perceptual function, *g*(*x*), is seen in Fig 3A. The concavity of the difference scaling function, *f*(*x*), in Fig 3B is consistent with diminishing returns as it indicates that the addition of the perception of smaller differences in *ψ* would overestimate the perception of a larger difference in *ψ*. Between the significant increase in predictive power and the concave difference scaling function, the responses from the in-person study support the existence of diminishing returns in the perception of achromatic stimuli.

### Comparison to crowdsourced results

To evaluate how well the crowdsourced data agree with the traditional experimental setup, we compared the in-person results with those from the crowdsourced experiment [22], whose code and data are publicly available. We compared the raw, crowdsourced responses and MLE analysis using triads centered at 50 and higher. We reran their analysis on this subset of their data, including cross-validation and bootstrapping.

**Fig 4:**
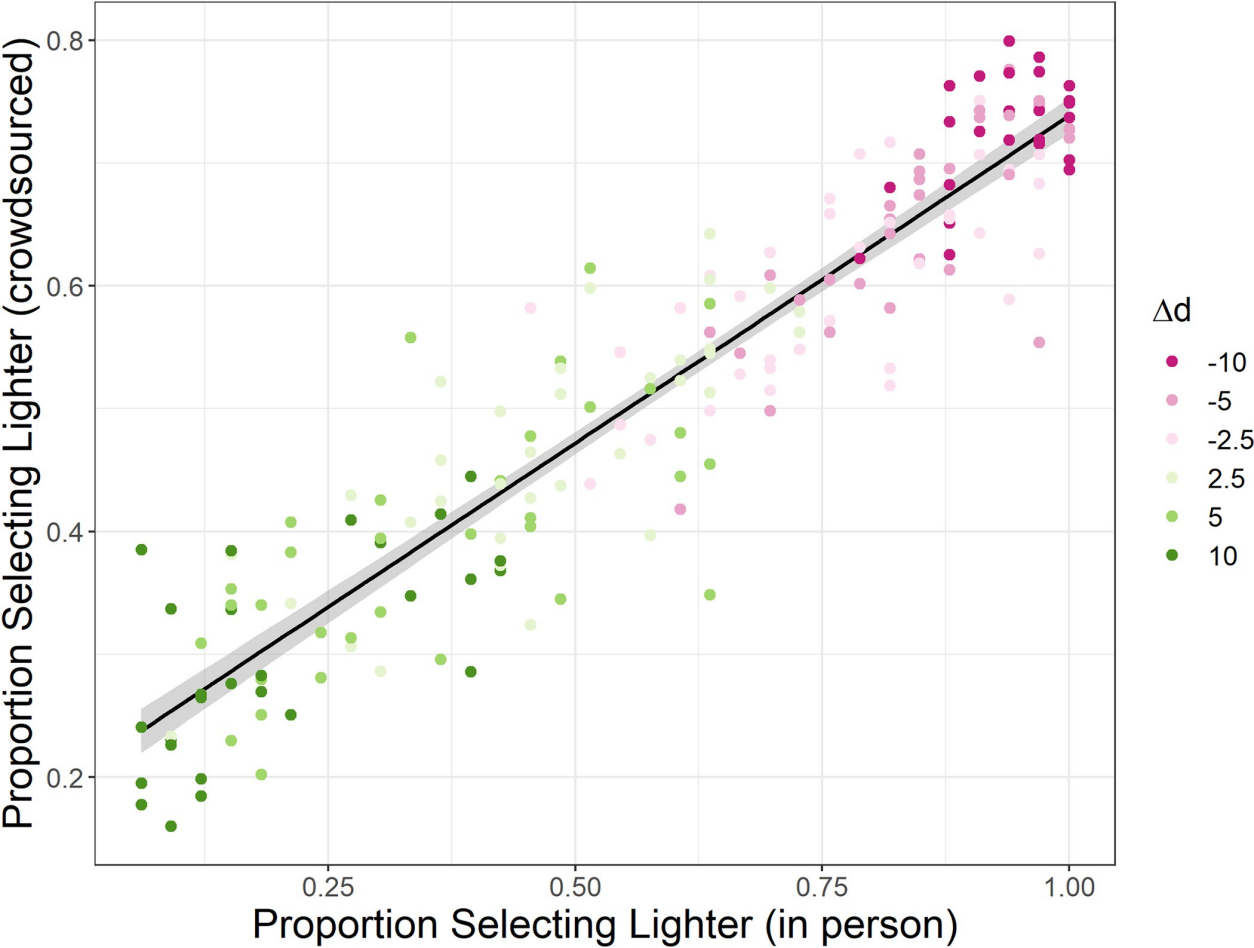
The proportion of participants selecting the lighter test based on which study they completed and the difference of differences.

First, the proportion of participants selecting the darker test in each triad is plotted based on the experimental setup in Fig 4. There was a significant, positive correlation, *R = 0*.*933*, *t(190) = 35*.*819*, *p < 0*.*001*.

**Fig 5:**
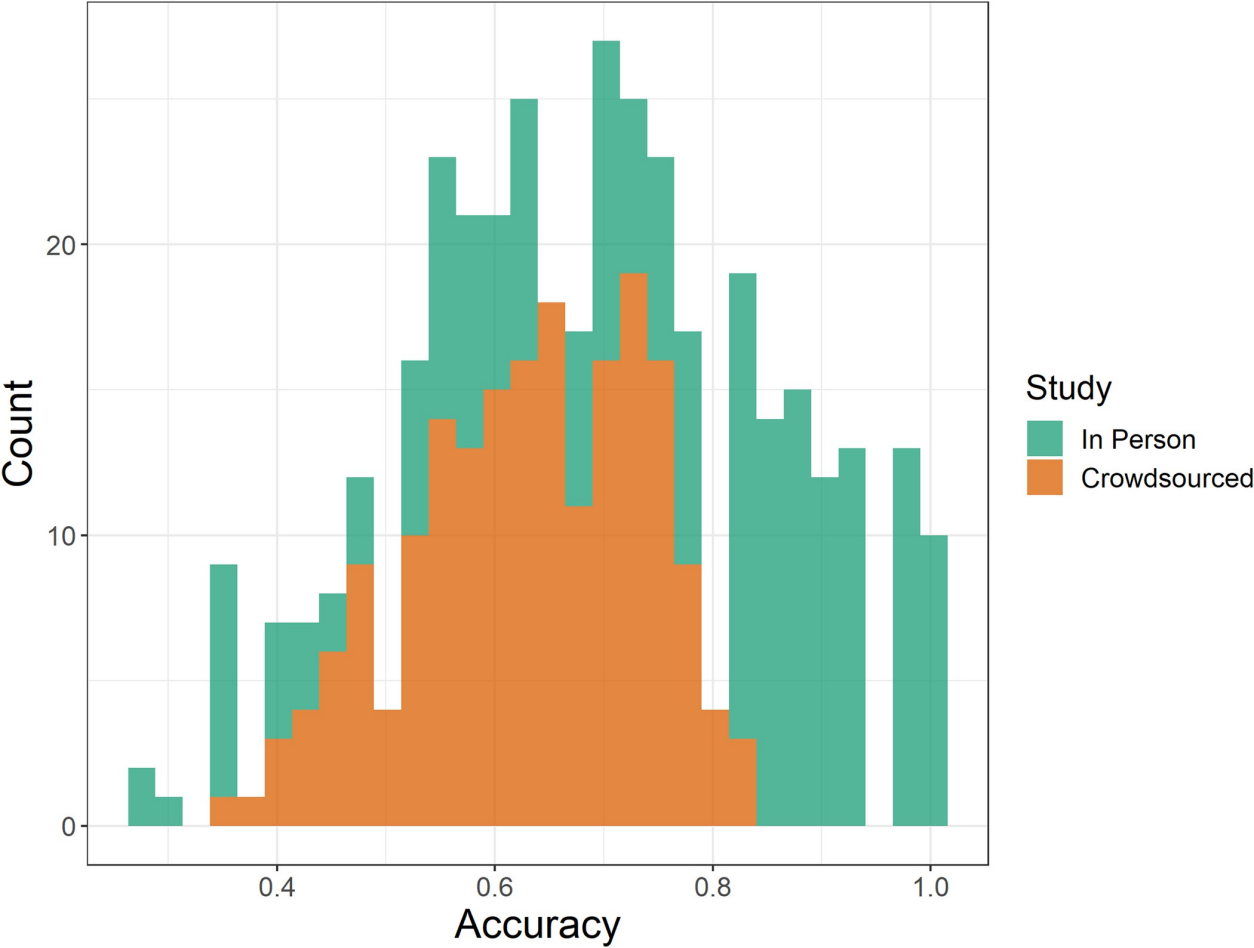
The histogram of accuracy per triad based on which study participants completed. Average accuracy for the in-person study was 74.2%, compared to the crowdsourced study, 63.0%.

In general, the accuracy of the in-person participants was higher than crowdsourced participants. We define accuracy based on if participants selected the larger difference using CIELAB coordinates. The accuracies based on triad are compared in Fig 5. This decrease in accuracy for crowdsourced participants is expected, as the uncontrolled setting in which participants completed the crowdsourced study likely artificially inflated the discriminal dispersion in the perceptual process.

**Fig 6:**
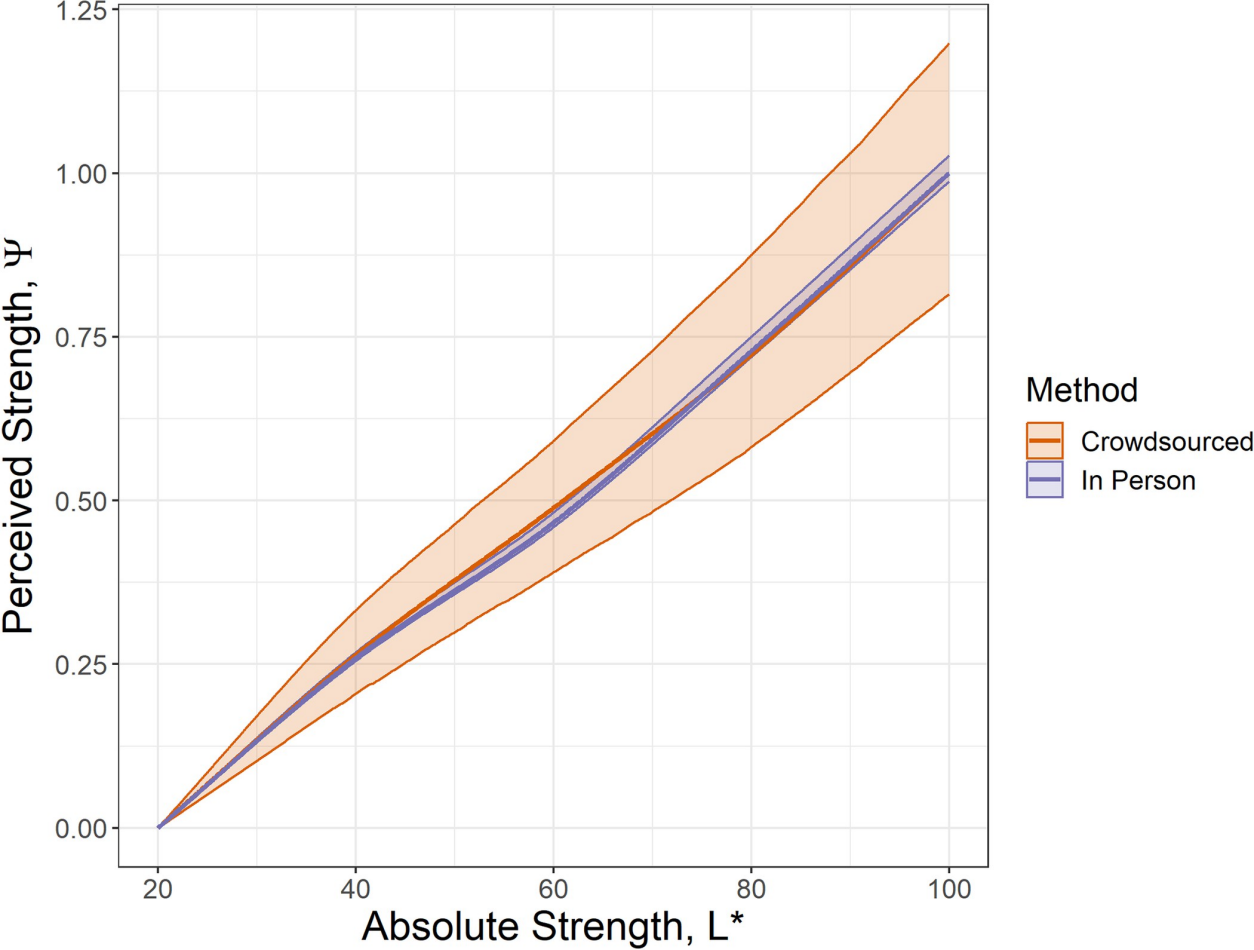
Learned perceptual functions, scaled so they share the range of.

The learned perceptual functions, *g*(*x*), were compared in Fig 6. The learned models were both scaled such that *ψ*∈[0,1]. The shaded regions around the plots correspond to the middle 95% of learned models. The crowdsourced model, despite coming from more data and using bootstrapping, has a wider margin of error, again consistent with more noise in the data due to the uncontrolled nature of the experiment. Despite the difference in experimental design, the perceived strengths fall in the same pattern.

**Fig 7:**
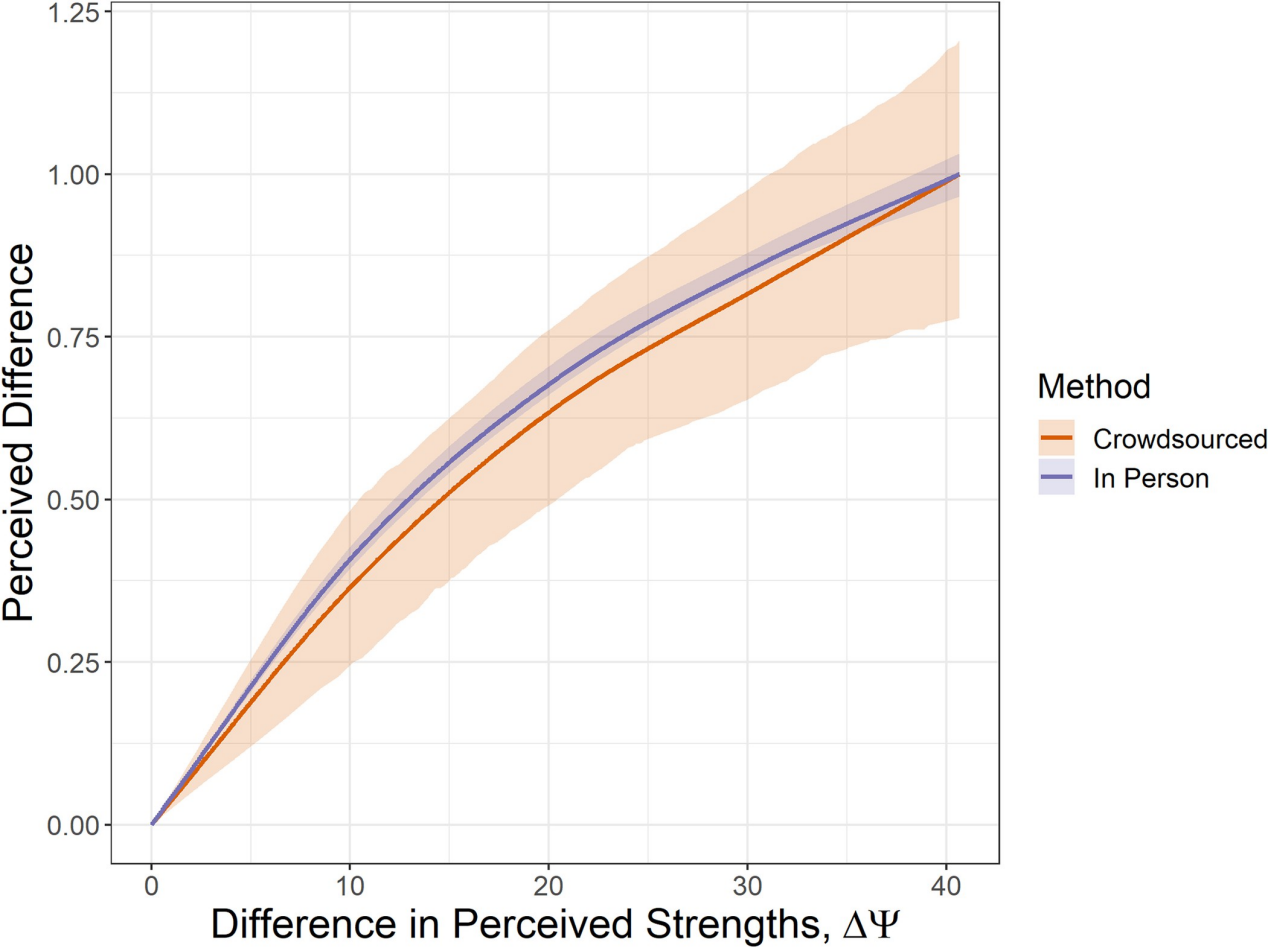
Learned difference scaling functions, scaled so they share both the domain and range.

The learned difference scaling functions, *f*(*x*), were compared in such a way that the domain and ranges are both shared. The resulting functions, see Fig 7, also follow the same pattern. The margins of error of the crowdsourced model encompasses the model from in-person participants, indicating that the model learned from the in-person data falls within the 95% confidence interval of the crowdsourced model. This suggests that the crowdsourced study was comparable to the in-person study.

## Conclusions

We successfully replicated a recent crowdsourced lightness perception study to determine if the noise attributed to reduced experimental control could be averaged out. Like we hypothesized, we found that the loss of control using a crowdsourced experiment can (a) be modeled statistically by a convolution of normally distributed variables and (b) overcome by increasing the sample size. In our case, we have shown that a sample size of approximately 300 participants was comparable to an in-person study of 34. Using Eq 7, we can conclude that the noise due to error is no more than approximately 3 times the discriminal dispersion. This is, to our knowledge, the first quantitative approximation of the noise due to lack of display calibration for achromatic stimuli on a color background, however it should be treated conservatively as it is for our specific stimulus presentation.

More importantly, we have shown that the Thurstonian model of perception applies to crowdsourced experiments as a convolution of normal distributions and derived a mathematical formula for *a priori* sample size determination when comparing crowdsourced experiments and in-person studies.

Also notably, we reproduced a crowdsourced study using an in-person paradigm, which to the best of our knowledge is the first time this type of reproduction is performed. There are plenty of studies replicating in-person studies with a crowdsource platform (see [4] for a review), however this direction is prone to inherent biases, namely the positive result selection bias where negative results are not published. Qualitatively, we saw support for diminishing returns, as in the previous study. Quantitatively, our learned models fell within the confidence intervals of the learned models from the crowdsourced study. Taken together, this supports the assumption that (1) the noise associated with crowdsourcing lightness perception studies are normally distributed and (2) can be averaged out with sufficient sample size.

Comparing the crowdsourced study and estimating the discriminal dispersion based on the analysis validation studies, we can be reasonably confident that the discriminal dispersion increased due to the uncontrolled nature of crowdsourced studies. The predictive power for the in-person model was notably lower than the crowdsourced study when using the combination of the perceptual function and the difference scaling function. Using responses from the in-person study, the average test accuracy was 66.4%, while the average test accuracy from the crowdsourced study was 91.6%. This decrease is likely due to a combination of the decreased data available for both training and testing, as well as the addition of bootstrapping in the crowdsourced study. The resulting estimations of the perceptual function and difference scaling function were not originally on the same scale due to the decreased overall participant accuracy in the crowdsourced study, but after correcting for that, the effect of diminishing returns was nearly identical.

We collected approximately one order of magnitude fewer responses in the highly controlled study (about 30 responses per triad) compared to the crowdsourced study (250 to 300 responses per triad). This difference in sample size between the two experimental setups is on the higher end when considering other comparisons of crowdsourced studies to in-person studies. Other comparisons with little to no difference in sample size used stimuli that were not expected to be significantly altered based on individual presentations in a crowdsourced environment (e.g. correlation in scatter plots [13, 14], graphical summaries [15, 16]). Demonstrating that even stimuli that are likely to suffer high noise due to crowdsourcing can be studied using an online platform with sufficient sample size is particularly impressive.

We expect our theoretical results to hold for different crowdsourcing platforms and different tasks even for studies in areas of psychology including and beyond color perception. For example, other crowdsourcing sites than MTurk have been shown to produce higher quality data, such as Prolific, [25]. Therefore, we expect the number of online participants needed to produce the same reliability of results to be less than those on MTurk, as the experimental noise is smaller for the stimulus character of interest.

This work was partially inspired by limitations on performing in-person studies during the Covid-19 pandemic. However, the implications of this work extend beyond pandemic-related restrictions. Crowdsourcing studies allows for a more representative sample, rather than recruiting students at institutions of higher education (sometimes referred to as the “Sophomore Bias”, [1–3], or Western, educated, industrialized, rich and democratic, WEIRD). Crowdsourced studies can also achieve a much higher sample size, enough to be more statistically powerful than traditional studies, and in a shorter time. Collecting the 34 participants for this study took approximately 2 weeks, while hundreds of crowdsourced participants can be recruited in a matter of hours.

The tradeoff in additional noise in crowdsourced experiments can be mitigated by an increased sample size to achieve sufficient statistical power. For example, consider a study investigating a difference of means using a perceptual judgment test. If the stimuli have an inherent discriminal dispersion of 5 arbitrary units (σ_d_ = 5) and an estimated noise factor from crowdsourcing the study of 12 arbitrary units (σ_n_ = 12), the needed sample sizes for an in-person study and a crowdsourced study can be calculated using *a priori* power analyses. G*Power allows us to easily perform this comparison using common values for ɑ (0.05), power (0.95), and an effect size of 2.5 for the in-person study [26]. The required sample size for the in-person version of the study (σ_in-person_ = 5) is 10. The comparable effect-size for the crowdsourced study (σ_crowd_ = √(5^2^ + 12^2^) = 13) is 0.96 and requires a sample size of 50. While a factor of 5 increase in sample size is certainly not negligible, recruitment on crowdsourced studies is much more efficient than in-person studies and collecting more than the required minimum sample size is highly feasible, thus increasing statistical power.

These findings suggest that crowdsourcing platforms, such as MTurk, can be used for lightness perception studies, as well as perceptual studies of color perception and in other areas of human perception, however the assertion that the noise is symmetrically distributed should be investigated further. The primary factor to consider is the relative noise due to lack of experimental control compared to the inherent discriminal dispersion. We have demonstrated this is possible for a lightness perception study, in which the lack of experimental control has a large impact on stimulus presentation, which supports the use of crowdsourced platforms for stimuli that would be just as impacted by lack of control and stimuli where the experimental control is less likely to have a dominating impact (e.g. scatterplot correlation). While crowdsourcing color perception studies raises reasonable skepticism, we report that such studies may be equally as effective as traditional studies with more representative samples. Crowdsourced studies do require larger sample sizes to overcome noise due to limited experimental control, but we have demonstrated that given the relative ease of collecting data on these platforms, an experimenter can achieve higher statistical power.
